# Reply to Saeed et al. Comment on “Rinaldi et al. Assessment of Response and Safety of Bulevirtide Treatment in Patients with Chronic Delta Virus Infection: The ARISTOTLE Pilot Observational Study. *Viruses* 2025, *17*, 251”

**DOI:** 10.3390/v17091228

**Published:** 2025-09-09

**Authors:** Alfredo Caturano, Luca Rinaldi, Antonio Izzi

**Affiliations:** 1 Department of Human Sciences and Promotion of the Quality of Life, San Raffaele Roma University, 00166 Rome, Italy; 2 Department of Medicine and Health Sciences “Vincenzo Tiberio”, Università degli Studi del Molise, 86100 Campobasso, Italy; 3 Department of Emergency Infectious Diseases and Infectious Diseases, Ospedali dei Colli, P.O.D. Cotugno, 80131 Naples, Italy; izziantonio@yahoo.it

We appreciate the thoughtful commentary by Muhammad Hassan Saeed et al. on our published article, “Assessment of Response and Safety of Bulevirtide Treatment in Patients with Chronic Delta Virus Infection: The ARISTOTLE Pilot Observational Study” (*Viruses* **2025**, *17*, 251) [1]. Their remarks raise important methodological considerations, which we address below.

Our study was intentionally designed as a real-world observational investigation to reflect the clinical context in which bulevirtide is currently used in Italy. It was not intended to function as a randomized controlled trial. The concomitant use of nucleos(t)ide analogs (NUCs), such as tenofovir or entecavir, is in line with current international and national treatment guidelines and represents the standard of care for patients with chronic HBV/HDV co-infection [2,3]. We acknowledge the concern regarding the absence of a bulevirtide monotherapy arm. In our clinical setting, the inclusion of an NUC-only comparator group was neither ethically nor practically feasible. The majority of patients with chronic HDV infection present with cirrhosis (89.5%), where treatment with NUCs is mandatory regardless of any detectable level of HBV-DNA to avoid disease progression towards end-stage liver disease [4]. The identification of NUC-only patients would thus be extremely rare, and they would represent a fundamentally different population compared to our cohort. A randomized trial excluding NUC in patients with cirrhosis would raise major ethical concerns; such a design could only involve patients without cirrhosis (a minority), and would inherently introduce survival bias, as these individuals generally have milder disease and better baseline status. Moreover, NUCs do not significantly reduce HBsAg levels, but only HBV DNA, and therefore do not directly affect HDV. Furthermore, clinical studies have shown that adding an NUC to bulevirtide provides no benefit in terms of virological response to HDV [5,6,7].

Bulevirtide was administered as an add-on therapy to these well-established regimens. While we recognize the possibility of confounding, the safety outcomes observed in our cohort were consistent with previously published data and did not reveal any unexpected adverse events [5], supporting the favorable tolerability profile of bulevirtide in routine clinical settings. Therefore, we strongly believe that currently, the use of randomized controlled trials, including monotherapy arms, to more precisely define bulevirtide’s standalone effectiveness and safety in clinical practice is completely unethical.

Regarding liver fibrosis assessment, we employed non-invasive modalities such as transient elastography, which have been widely validated in the field of hepatology and currently represent the standard of care in clinical practice worldwide, offering a simple, feasible alternative to invasive procedures and one that is well accepted by patients [8]. Although liver biopsy remains the gold standard, its use in observational studies poses significant limitations, including ethical concerns, procedural risk, and patient reluctance [9]. In this context, our approach reflects a pragmatic and ethically appropriate strategy for data collection in real-world settings. Moreover, in our real-world experience, several patients were presenting with clinically significant portal hypertension, represented by thrombocytopenia, esophageal varices, and neutropenia, thus rendering liver biopsy of limited utility in clinical practice. We acknowledge the value of complementary fibrosis scoring methods and will consider histological or standardized scoring tools in future prospective studies. In the current analysis, we incorporated non-invasive indices including FIB-4 and APRI scores alongside elastography, which provided additional insights into fibrosis severity.

On the topic of vitamin D supplementation, we acknowledge this as a potentially relevant variable. However, data on vitamin D supplementation were not systematically collected at study initiation and were available for only a limited number of patients. As such, statistical analysis regarding its influence on treatment response was not feasible. Recognizing this limitation, we have incorporated vitamin D supplementation data into the ongoing extended phase of the study to facilitate more robust evaluations in future analyses. We therefore concur that these observations are best classified as exploratory and hypothesis-generating. Notably, in our multivariable logistic regression analysis, vitamin D levels emerged as a potential predictor of improved virological response, warranting further investigation in larger, systematically collected datasets.

We also acknowledge the limitation posed by our relatively small sample size and the absence of predefined treatment stratification, but we emphasize that they are still preliminary data. On the other hand, it is important to emphasize that the patient data were derived from 13 tertiary referral hospitals and academic hepatology centers across Italy, which collectively manage a substantial proportion of the national HDV patient population. As a result, our study reflects real-world management and best clinical practice outcomes in high-volume, expert clinical settings, offering valuable insights despite the sample size constraints. To address these issues, we are currently engaged in an expanded data collection phase involving a larger, multicenter cohort with longer follow-up. This effort is aimed at validating and refining our initial findings by improving statistical power and enabling more granular subgroup analyses. We fully agree that future studies should strive for larger, well-characterized cohorts, predefined treatment arms, comprehensive reporting of concomitant medications and supplements, and, where feasible, histological confirmation of liver fibrosis.

We conclude that bulevirtide remains a very promising therapeutic option in real-world management of chronic HDV, and larger, well-designed studies will further define its optimal role.
